# CIRSE Standards of Practice on Bronchial Artery Embolisation

**DOI:** 10.1007/s00270-022-03127-w

**Published:** 2022-04-08

**Authors:** Joachim Kettenbach, Harald Ittrich, Jean Yves Gaubert, Bernhard Gebauer, Jan Albert Vos

**Affiliations:** 1 Landesklinikum Wiener Neustadt, Institute of Diagnostics, Interventional Radiology and Nuclear Medicine, Wiener Neustadt, Austria; 2 Department of Diagnostic and Interventional Radiology, Schoen Clinic Hamburg Eilbek, Hamburg, Germany; 3 Department of Radiology, Timone University Hospital, Marseille, France; 4 Laboratory of Experimental Interventional Imaging, Aix-Marseille University, Marseille, France; 5 Department of Radiology, Charité-Universitätsmedizin Berlin, Berlin, Germany; 6 Department of Interventional Radiology, St Antonius Hospital, Nieuwegein, Utrecht The Netherlands

**Keywords:** Bronchial artery, Embolisation, Endovascular treatment, Haemoptysis, Life-threatening haemoptysis, Massive haemoptysis

## Abstract

This CIRSE Standards of Practice document is aimed at interventional radiologists and provides best practices for performing bronchial artery embolisation to effectively treat haemoptysis. It has been developed by an expert writing group established by the CIRSE Standards of Practice Committee.

## Introduction

The CIRSE Standards of Practice Committee established a writing group, which was tasked with producing up-to-date recommendations for performing bronchial artery embolisation (BAE). This document is not a clinical practice guideline or a systematic review of the literature. As with all CIRSE Standards of Practice documents, this document is not intended to impose a standard of clinical patient care, but recommend a reasonable approach to and best practices for performing BAE.

Institutions should regularly review their internal procedures for development and improvement, taking into account international guidance, local resources and regular internal morbidity and mortality reviews.

A summary of key recommendations for BAE can be found in Table 1.

**Table 1:** Summary of key recommendations

In patients with life-threatening haemoptysis, multidetector computed tomography (MDCT) and computed tomography angiography (CTA) are the first diagnostic tests, except for patients in whom bleeding must be controlled and the airway ensured; in which case bronchoscopy will be the first test
Carefully scrutinize angiography for any signs of non-target vessels leading to brain, spine cord and heart before starting the embolisation procedure
Use the left main bronchus as a reliable reference point for catheterisation of the right or left bronchial arteries
Control haemoptysis in chronic cavitary pulmonary aspergillosis (CPA) by combining tranexamic acid and BAE
Non-spherical, polyvinyl alcohol (PVA) particles, 355–500 µm in diameter are recommended for BAE
Never use particles with a diameter < 300 µm for BAE
Consider calibrated spherical microspheres > 300 µm in diameter and NBCA as an alternative to PVA-particles for BAE
In case of bronchial-to-pulmonary shunts, use larger particles and coils
Actively search and embolise as many non-bronchial systemic collaterals in the first BAE procedure as possible to decrease recurrence rates of haemoptysis
In addition to BAE treatment of underlying disease such as in aspergillomas, and multi-drug resistant tuberculosis (TB) is required

## Methods

The writing group for this document, which was established by the CIRSE Standards of Practice Committee, consisted of five clinicians with internationally recognised expertise in bronchial artery embolisation. The writing group reviewed existing literature on BAE, performing a pragmatic evidence search using PubMed to search for relevant publications from 1974 to 2021. The writing group formulated the recommendations by consensus.

## Background

Severe haemoptysis (SH) is a respiratory emergency, associated with a high mortality rate of 50–100% [1]. The cause of death is usually asphyxiation and not exsanguination. However, when optimal diagnosis and treatment are provided, mortality is less than 20% [1].

First described by Remy et al. [2], various algorithms and techniques for BAE have been reported [1, 3–9]. In 90% of massive haemoptysis cases, the culprit vascular bed is the bronchial circulation rather than the pulmonary (5%) or non-bronchial systemic circulations (5%) [3].

Frequencies of the aetiologies (Table 2) vary greatly depending on the geographic origin, on the location of treatment and the severity of the haemoptysis [1].

**Table 2:** Major causes of massive haemoptysis [1, 11, 14]

Tuberculosis (active and inactive)
Bronchiectasis (TB, cystic fibrosis, other)
Abscess
Chronic pneumonia
Aspergilloma (isolated or with pre-existing chronic lung disease, e. g. sarcoid, TB)
Lung carcinoma (including metastases)
Chronic pulmonary interstitial fibrosis
Chronic obstructive pulmonary disease (COPD), chronic bronchitis
Pneumoconiosis
Pulmonary artery malformation (PAVM), Rasmussen or mycotic aneurysm
Cryptogenic haemoptysis (preceding malignancy in 10%)
Anticoagulant treatment

In developed countries, the most prevalent aetiologies of haemoptysis are, in 80% of cases, lung cancer, active tuberculosis and its sequelae, bronchiectasis, and aspergillosis [10, 11]. No aetiology is identified in 15–20% of cases [1]. In India and Turkey, tuberculosis (60–90%) is the main aetiology of SH [1].

## Indications for Treatment and Contraindications

Embolisation is indicated in all patients with life-threatening or recurrent haemoptysis (Table 3) in whom pathological arteries are observed [3, 6, 12, 13]. BAE may also serve as a bridge treatment for patients with moderate or severe haemoptysis caused by chronic inflammatory lung diseases such as cystic fibrosis (CF), who qualify for lung transplantation [4]. BAE is also recommended in sudden onset of blood-streaked sputum followed by mild episodes of haemoptysis with increasing volumes. Such smaller “index-bleeding” often precede life-threatening haemoptysis (LTH) [3].

**Table 3:** Indications for bronchial artery embolisation [1, 6, 9, 15]

Any haemoptysis causing significant airway compromise or respiratory distress
Three or more episodes of haemoptysis with 100 ml blood or more within 1 week
Chronic or slowly increasing bleeding episodes

The only absolute contraindication is a supplying branch to the heart, brain or spinal cord (Table 4). All other contraindications are relative and the risk of not treating massive haemoptysis generally outweighs any risks [14].

**Table 4:** Contraindications for bronchial artery embolisation [4, 14]

*Absolute contraindication* Supplying branches to the heart, spinal cord or brain arising from bronchial, intercostal or other non-bronchial vessels
*Relative contraindication* Congenital pulmonary artery (PA) stenosis (bronchial collateral vessels may provide an essential role in pulmonary parenchymal perfusion)

## Patient Preparation

### Pre-procedural Evaluation and Imaging

#### Assessment of Haemoptysis

Suspected haemoptysis must be confirmed (see grading in chapter definition), its severity established, the origin of bleeding located and the cause determined. Risk factors (infections, malignancies, cardiac disorders, vasculitis, collagenosis, coagulation disorders), medications (especially anticoagulants), traumatic pulmonary injury or iatrogenic causes (biopsy, recent Swan-Ganz catheter use) should be considered.

#### Laboratory

Clinical laboratory tests include a complete blood count, coagulation parameters and biochemistry. Pulse oximetry and arterial blood gases to determine the impact of haemoptysis on oxygenation and ventilation. An aspergillus precipitin test may help to detect pulmonary aspergillosis. Mantoux in patients with suspected tuberculosis, and blood cultures or serology if infectious disease is suspected [15].

### Sputum Examination

Obtain a cytological study and sputum microbiology to establish the presence of bacteria (Gram stain, potassium hydroxide, and acid-fast bacillus), mycobacterium and fungi [15].

### Chest Radiograph

Chest radiograph (CR) may help in diagnosing and localising an underlying source of haemoptysis but is known to have limited sensitivity [16, 17]. Comparison of a CR with previous films may be helpful, as new changes indicate the side to be assessed first.

### Multidetector Computed Tomography

Multidetector CT (MDCT) scan is superior to chest radiograph to identify both the anatomic origin and underlying cause of haemorrhage and to define the course of bronchial and non-bronchial systemic arteries [16, 18–21].

A non-contrast-enhanced MDCT (sufficient on patients with blood-streaked sputum and suspected bronchiectasis) identifies the cause and location of bleeding in 82–100% of cases [15, 22].

Patients with blood-streaked sputum and risk factors (smoker, COPD) or with pathological findings on chest X-ray require a MDCT with intravenous contrast medium [15]. Patients with SH and active bleeding require an angio-MDCT, from the base of the neck to the renal arteries [15] to identify a non-bronchial arterial supply before BAE [1].

Pleural thickness of more than 3 mm and enlarged vascular structures within extrapleural fat (Table 5) are good indicators that a non-bronchial systemic arterial supply is the cause of bleeding [23]. MDCT may also localise other pathologies (Table 2) responsible for haemoptysis [24].

**Table 5:** Angiographic and CT appearance of abnormal bronchial arteries [9]

Hypervascularity of lung parenchyma (most common)
Hypertrophic tortuous bronchial or non-bronchial arteries (common)
Neovascularisation (common) or peri-bronchial hypervascularity
Enlarged main bronchial artery (diameter > 2.0 mm)^a^
Contrast extravasation (variable)
Bronchial artery aneurysm, pseudoaneurysm (rare)
Bronchial-to-pulmonary vein-shunts
Pleural thickening > 3 mm adjacent to a parenchymal abnormality
Extrapleural fat hypertrophy including enlarged vascular structures

^a^In a healthy subject, the diameter of the bronchial artery at the ostium measures 1.6 ± 0.3 mm with a range of 1.1–3.0 mm [56]

MDCT is limited in characterising endobronchial blood clots that may mimic tumours, or when blood clots mask the presence of an endobronchial process. However, the combination of both bronchoscopy and MDCT was diagnostic of the aetiology of haemorrhage in 84% of cases [1, 20, 22].

**Fig. 1:**
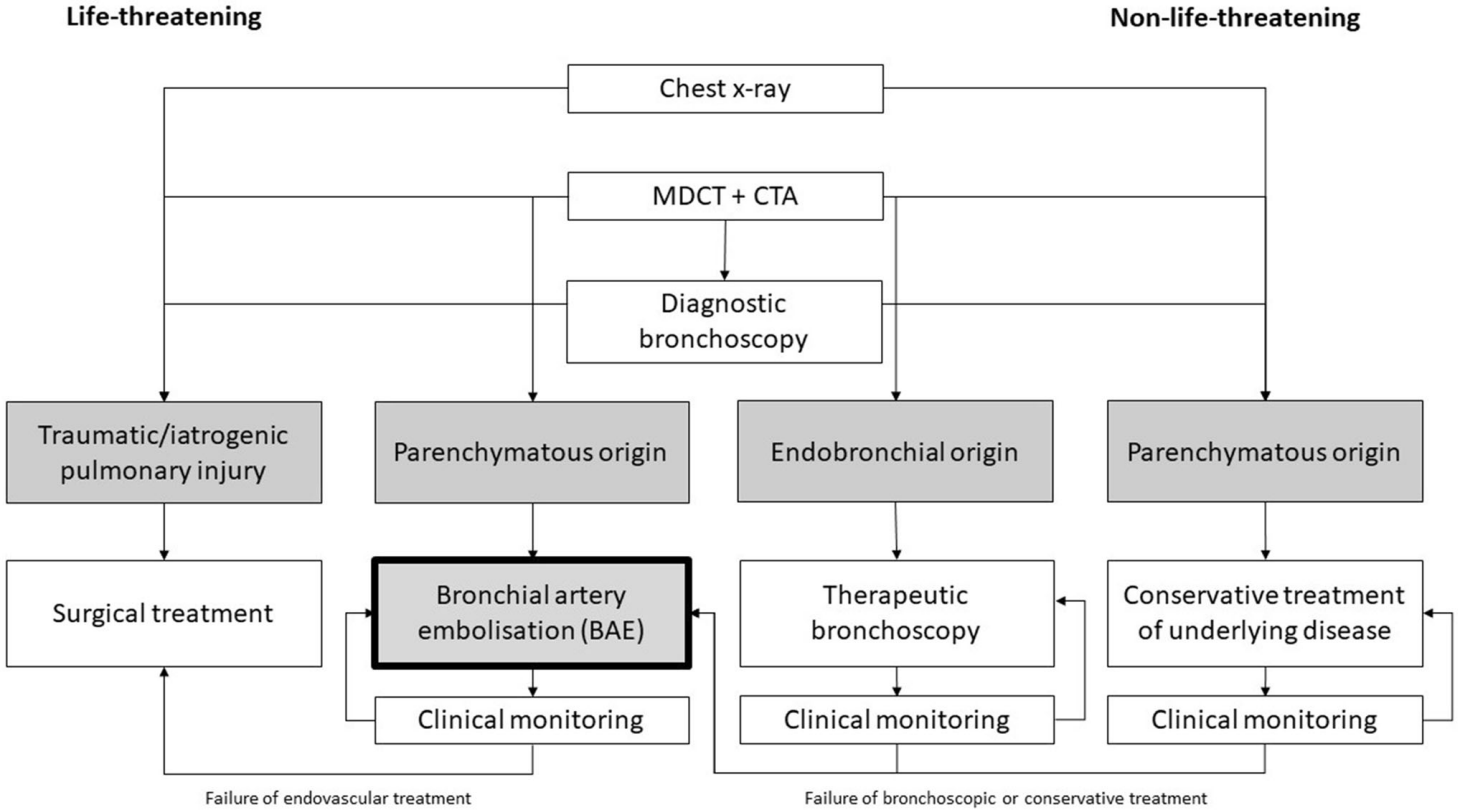
Flowchart of diagnosis and treatment of haemoptysis (modified from [60]). *MDCT* multidetector computed tomography, *CTA* computed tomography angiography

Except for life-threatening situations, MDCT is performed before bronchoscopy (Fig. 1) [16].

### Bronchoscopy

Bronchoscopy (BC) is used for lateralisation, allows clot extraction, direct instillation of medications, tamponade and ablation of haemorrhagic arteries and supports selective intubation to maintain airway patency [20]. The source of active bleeding is most likely to be located when bronchoscopy is performed during active haemoptysis (in 73–93% of cases) or within 24–48 h of cessation [25].

In patients who are unstable, where transfer is unsafe and intubation and lung isolation is of the utmost importance, flexible bronchoscopy (FB) is the procedure of choice and can be performed at the bedside [1, 15, 20]. In addition to the visual examination, FB is used to collect samples for cytology and microbiology.

Rigid bronchoscopy provides quick evacuation of large obstructing blood clots and superior airway stabilisation, but requires an operating room and general anaesthesia, and does not visualise the distal airways. However, rigid bronchoscopy in combination with FB is the safest and most comprehensive procedure in LTH [15].

If bronchoscopy is not readily available, it should not delay care to a patient who is clinically stable for transfer to CT scan for efficient haemorrhage localisation and a speedy transition to definitive therapies such as BAE [20].

### Digital Subtraction Angiography

Digital subtraction angiography (DSA) is indicated where endovascular treatment has to be attempted and other diagnostic studies, such as bronchoscopy or MDCT, have already been completed [1].

The flowchart in Fig. 1 details the diagnosis and treatment of haemoptysis.

## Treatment

### Equipment Specifications

Adequate, high-resolution digital subtraction angiography (DSA) is a prerequisite.

#### Sheath and Catheter

For access via the right common femoral artery, a long 5-Fr. introducer sheath (45 cm) is preferred in patients with tortuous arteries to negotiate the effect of the curves on catheter manipulations [19]. Transbrachial or transradial access can be used as well and may be required to catheterise aberrant vessels from the subclavian artery or the inner curve of the aortic arch [4, 24].

The selection of guidewire and diagnostic catheter (DC) depends on the anatomy (Table 6). End hole-only design is essential and 4-Fr. or 5-Fr. Sidewinder or Mikaelsson catheters allow a more stable ostial position than forward facing cobra-curved catheters. A diameter of 4 Fr. carries a lower risk of damaging the BA ostium.

**Table 6:** Equipment for bronchial artery embolisation

Diagnostic Catheters
Reversed curved shapes
Forward-looking shapes
Microcatheter
2.7–2.9 Fr. preferred
Microwires
0.014″ or 0.016″ in diameter, hydrophilic coating preferred
Non-ionic contrast
Embolic agents
Spherical or non-spherical particles (300–900 µm)
Liquid embolic (NBCA, non-adhesive, high-viscous polymers)
Gelfoam (only as supplementary agent)
Microcoils (in specific situations)

A braided, hydrophilic low-profile 1.9–2.8 Fr. microcatheter (MC), with internal diameter of 0.0165–0.027 inch is preferred as coaxial “tapered” extension of the diagnostic catheter for super-selective catheterisation [6]. Continuous flushing of the DC by a drip infusion using a haemostatic valve may be advantageous.

#### Contrast Media

Non-ionic contrast agents will minimise the risk of transverse myelitis [4].

#### Embolic Materials

Non-spherical, PVA particles, 355–500 µm in diameter were most commonly used [6, 14, 21, 26, 27]. Due to their non-uniform shape, PVA particles can aggregate and form plugs that result in premature embolisation proximal to the intended level [28]. Occlusion is completed by thrombus formation and moderate perivascular inflammatory change [29].

Alternatively, any calibrated microsphere particles (300–900 μm) can be used [6, 30]. These particles are more uniform in size and in penetration characteristics than PVA, and their smooth hydrophilic coated surface is less prone to clumping within catheters [27, 29, 30].

When large pulmonary arterial or venous shunts are present, larger sized tris-acryl microspheres (700–900 μm) or coil embolisation may help avoid complications [14, 31, 32] such as pulmonary, myocardial, or systemic infarcts [13, 32]; the presence of spinal cord feeders arising from the bronchial artery also seems to be less critical when using larger sized (700–900 μm) particles [19, 27].

However, microparticles with a larger diameter may occlude the index bronchial artery more proximally than preferred, which could lead to recurrent haemoptysis from systemic collaterals. This risk can be diminished by placing the tip of the MC to a location as close as possible to the abnormal vasculature [27].

Smaller particles (< 300 μm) may occlude regular blood supply of the bronchi, oesophagus, vasa vasorum of the pulmonary artery or the aorta, with increased risk of excessive tissue ischaemia and necrosis and therefore may not be used in BAE [33].

Occasionally small quantities of gelatine in the form of a pledge or thick slurry can be placed after particulate agents to complete the embolisation, but its use as a sole agent is not as durable, and thus not recommended [13, 22].

N-butyl cyanoacrylate (NBCA) glue mixed with iodised oil for both opacity and modification of the polymerisation rate has shown a better haemoptysis control rate than PVA [13, 28]. A variable degree of vessel penetration is controlled by the injection rate and the dilution ratio at the time of preparation, which is typically 1:2 to 1:4 NBCA to lipiodol [28]. Inexperience with NBCA may potentially lead to a variety of pitfalls, including premature polymerisation, non-target embolisation, or attachment of the MC tip to the glue cast. NBCA is not recommended for the treatment of haemoptysis until the provider has gained significant familiarity with its behaviour in other settings [13].

Non-adhesive, high-viscous polymers dissolved in dimethyl sulfoxide (DMSO) include tantalum powder or iodine component to enhance visibility and contrast [1, 34] in cases of refractory massive haemoptysis in patients with CF where conventional particle embolisation has failed [35]. However, similar to NBCA, application of liquid polymers requires experience, and aggregation of the polymer may block the MC as well.

The prevailing view of using microcoils for BAE is that coil embolisation would prevent future BAE for recurrent haemoptysis due to proximal embolisation and the development of challenging collateral vessels [14, 22, 36]. However, high-packing-density coil deployment, using hydrogel-polymer-coated or high thrombogenic coils may be still used in recurrent haemoptysis [37] to protect spinal supply [31] and distal circulation, to occlude actively bleeding vessels as in pseudoaneurysms, or to occlude large bronchial-to-pulmonary shunts [3, 5].

Self-expanding microplugs (diameter 5.0–6.5 mm) loaded within a 0.027″ microcatheter can occlude blood flow within BAs 1.5–5 mm in diameter [38]. To our knowledge, only one study reported a vascular microplug system to treat paediatric haemoptysis; however, the procedure had to be repeated up to 4 times to stop haemoptysis [39].

For further information about current available microcatheter and embolic agents, one may consult the European Device Guide website [40].

### Procedural Features and Variations of the Technique(s)

In the absence of a preceding contrast-enhanced MDCT, a flush aortogram can be obtained to identify bronchial and non-bronchial systemic collaterals [5, 6]. A shallow left anterior oblique angulation may help to visualise the origins of bronchial arteries. The field of view should extend several centimetres superior to the lung apices to avoid exclusion of important apical collateral vessels.

Angiography of the subclavian arteries should be considered, to determine the origin of ectopic BA supplied by internal thoracic arteries or thyrocervical trunks, in apical-predominant disease [24]. When the origin of the BA has been documented by the preprocedural CT, the procedure will start with the selective catheterisation of the BA. After careful control of the DC stability, selective angiogram of the BA will be performed–hand injection will suffice for adequate opacification in most cases [3].

The DC is then gently advanced 1–2 cm through the ostium of the BA, avoiding complete occlusion of the lumen. Rigorous verification of absence of any branches supplying critical structures, such as the spinal cord, is mandatory before commencing BAE. These branches may arise directly from intercostal bronchial trunks, or may be in close communication via short collateral vessels.

The MC should not be advanced too far in the target vessel, in order to preserve free flow around its distal tip. Any “aggressive” manipulation of the guidewire may induce spasm, dissection, or rupture of the BA [1].

Immediately before embolisation, a hand injection of contrast through the MC using a small size Luer-lock syringe should be performed, at a rate determined not to cause reflux [33]. Note that this rate will change during embolisation, as downstream flow resistance increases.

At all times embolisation is performed with a small (1 ml) Luer-lock syringe under active fluoroscopy to ensure any sign of reflux is picked up as early as possible. Careful pulsatile injection of minute amounts of the embolic agent may be slowly continued when there is no resistance to the blood flow. When the resistance builds up or a limited reflux appears, a new manual contrast injection has to be carried out after carefully flushing residual embolic material through the catheter. Vigilant attention to newly appeared collateral vessels, to bronchial-to-pulmonary shunts or reverse flow to branches supplying the spinal cord, or other vital structures is mandatory.

When flow is sluggish, stop embolisation and carefully flush the remaining embolic material within the MC with saline. If flow has already stopped, flushing the MC should be avoided and aspiration of embolic agents within the MC lumen is necessary until free-flowing blood is obtained. If unsuccessful, removal of the MC during aspiration and flushing outside the patient is recommended.

When bronchial branches originate from the aortic arch, mammary or subclavian arteries, careful consideration should be given during embolisation, since tiny amounts of reflux can cause cerebral emboli.

For good haemoptysis management, all pathological arteries must be embolised [15]. In malignant disease, the aim is to obtain permanent occlusion of the abnormal circulation distally. In chronic inflammatory disease such as CF, reduction of blood flow will sufficiently reduce the vascular pressure, thus lowering the risk of recurrent haemoptysis [13].

Do not inject intercostals at the vessel origin as this increases the chance of radiculomedullary branch embolisation. Rather advance the MC a few centimetres into the vessel (at least beyond the pedicle) to avoid particle reflux [14].

Where clinical or imaging lateralisation of the bleeding site is uncertain, treat any enlarged bronchial arteries at the first session. If no bronchial supply or abnormality is detected, consider MDCT to exclude bleeding sources such as pulmonary artery aneurysms, PAVMs and fistulas [1, 3, 41]. When the culprit lesion is not identified, or if there is any abnormal circulation on both sides, bilateral BAE is required.

An angiogram of lower thoracic and abdominal aorta may help to detect the origin of vessels arising from phrenic arteries or other abdominal branches (in cases involving the lower lobes).

### Surgery

Open surgical options must be undertaken urgently in cases in which haemorrhage of the pulmonary arteries is caused by a destructive pulmonary process (lung cancer, necrotising pneumonia, pulmonary mycetoma) [15] or if other methods fail [20].

## Medication and Peri-Procedural Care

As long as no immediate intervention is necessary such as in life-threatening haemoptysis, clotting parameters and haemodynamic status will be optimised prior to intervention [31] and fasting of 3–6 h (depending on local protocol and type of sedation/anaesthesia) is recommended in elective cases to prevent aspiration.

Ensure the placement of large-calibre venous access for fluid management, and if required, availability of red blood cell transfusion [15]. Monitor the vital parameters (pulse-oximetric oxygen saturation (SpO_2_), respiratory and non-invasive blood pressure measurement. During the procedure, apply supportive measures (oxygen 3 l/min through the mask, bronchodilators, hydration, etc.). Administration of antitussives controls coughing.

BAE requires a co-operative patient and skilled airway management is essential [3]. Thus, generous support by anaesthesia is recommended [6]. Patients with chronic lung disease and dyspnoea can significantly worsen in a horizontal position during angiography. If the patient is not intubated, consider inclination of the upper body by tilting the angiographic table accordingly.

If the patient cannot eliminate blood from the tracheobronchial tree or if respiratory failure is severe, help the patient with the affected lung dependent to decrease overspill into the non-bleeding lung. If the patient is unstable, start with single-lung ventilation first and bronchoscopy must be performed as soon as possible after intubation (Fig. 1).

If BC is not available, a CR followed directly by DSA including BAE would likely be the best approach [15].

## Post-treatment Follow-Up Care (Including Imaging)

An arterial closure device at the puncture site allows patients with respiratory compromise a more comfortable sitting position. Immediate post-procedural care includes haemodynamic monitoring and checking the arterial access site.

Observe for signs of neurological deficit [4, 14]. Provide supplemental oxygen, IV fluids, and blood pressure maintenance (mean arterial pressure above 80 mmHg) if spinal cord damage occurs. A spinal drain should be inserted if spinal cord infarction cannot be immediately confirmed [14].

Ensure cessation of haemoptysis during follow-up [42]. In the event of recurrent haemoptysis, consider non-bronchial systemic arterial supply to the lung.

Most patients with chronic pulmonary disease, such as CF, will require repeat embolisation every 2–3 years, as new collateral vessels open up. However, these embolisations become increasingly challenging, and carry increasing risk of non-target embolisation as more collaterals develop [14].

Antifibrinolytics (aminocaproic acid, or tranexamic acid) may be helpful in minor recurrent haemoptysis [1, 15, 31, 43]; however, there is too little evidence to judge whether any antifibrinolytics should be used to treat haemoptysis [43].

BAE is merely a symptomatic therapy without eliminating the underlying cause. Thus, a causal therapy must always follow a successful BAE to ensure the long-term success of embolisation. When effective embolisation is impossible and bleeding recurs within 72 h of completing the procedure, emergency surgery should be considered [15].

## Outcome of BAE in Haemoptysis

### Technical Success

Technical success is defined as the ability to catheterise and embolise the abnormal bronchial or non-bronchial arteries that are responsible for the bleeding [44]. Technical success rates ranging from 90 to 100% have increased due to the development of more meticulous techniques and superselective embolisation [9, 45, 46]. Technical failures are usually caused by inability to achieve a secure and stable catheter position in BA; failed embolisation in extensive and bilateral disease; or not recognizing the pulmonary artery as an origin of the bleeding.

### Clinical Success

Clinical success, defined as complete cessation of haemorrhage or significant reduction in haemoptysis after BAE without requiring further intervention for at least 24 h or within 30 days, was 82–100% [1, 5, 6, 47] and 70–92%, respectively [9, 27, 47] (Table 7). However, the rate of recurrent haemoptysis (up to 47% [42]) is still quite high [9, 48] and was associated with significantly increased mortality (*p* = 0.021) compared to patients without recurrence [42].

**Table 7:** Success rates for percutaneous transcatheter embolisation [8, 9]

	Reported success rates (%)
Bronchial artery embolisation	
Initial technical success (all indications)	90–100
Clinical Success	
within 24 h after BAE	82–100
within 30 days after BAE	70–92
1-year clinical success (all indications)	64–92
Aspergillosis and malignancy	58–67
Aspergillosis, immediate success	64
Aspergillosis, recurrence of haemoptysis	52
Aspergillosis, repeated BAE after first BAE	76
Cystic fibrosis	
Clinical success	95
9-month success	64–68

Reported technical and clinical success rates are categorised according to the guidelines for percutaneous transcatheter embolisation established by the Society of Interventional Radiology Standards of Practice Committee [45]

Predictors of recurrent haemoptysis (Table 8) were as follows: recruitment of non-bronchial systemic collaterals [3, 6], diabetes [8], presence of an aspergilloma [8], feeding vessels from internal mammary artery [8], multidrug-resistant tuberculosis, co-existent pulmonary interstitial lung disease, patients with malignant diseases [27], unstable haemodynamics and prolonged coagulation [9, 42].

**Table 8:** Short- and long-term risk factors for recurrence of haemoptysis following BAE [42]

Incomplete embolisation
Inadequate treatment of underlying disease
Progression of underlying disease
Recanalisation of embolised vessel
Recruitment of non-bronchial systemic arterial supply
Use of resorbable agents (gelfoam)
Use of coils only
Use of systemic vasoconstrictors medication before BAE
Vessel spasm
Lack of operator experience (less than 5–10 cases/year)
Patients with aspergilloma
Received blood transfusion at the time of BAE
No cessation of haemoptysis within 7 days

The long-term success rate can be improved with repeat BAE of recurrent haemoptysis, particularly in CF patients [49], or by embolisation of ≥ 2 vessels [9]. Risk for relapse of haemoptysis was significantly lower for patients with bronchiectasis as compared with other chronic infections (*p* = 0.0022) and cystic fibrosis (*p* = 0.0004) [46]. Also, a protective effect of anti-tuberculosis treatment was observed in patients treated with BAE [42].

BAE with NBCA provided significantly higher haemoptysis-free survival rates at 1, 3 and 5 years as compared with PVA particles [28]. The difference in level of embolisation may explain these results.

Adults with CF bear a much higher risk of respiratory function deterioration after BAE [50]. A superselective embolisation occluding only the most likely culprit vessel reduces this risk even in CF patients with severely compromised pulmonary function [13].

Comparing PVA particles (355–710 μm in diameter) vs. gelatine cubes (1 mm^3^), a significant higher percentage of patients were free of haemoptysis at 12 months using PVA (*p* = 0.02 and *p* = 0.03, respectively) [26]. Using trisacrylic microparticles of 700–900 µm diameter, the long-term recurrence rate was as low as 8.1% with a follow-up period of up to 56 months [27].

### Procedure-Specific Complications

Major complications of BAE such as spinal cord infarction, once reported in up to 6% [28, 48], are far less common (< 1%) in recent series [13, 48].

Rare reported major complications include bronchial infarction [51], oesophago-bronchial fistula, myocardial [52] or spinal cord infarction [48, 52], ischaemic colitis [53], transient cortical blindness, and stroke [30, 50, 54], all of which can be attributed to non-target embolisation [3].

Minor complications include transient chest pain (in up to 91%) and dysphagia [13], both usually self-limiting, but symptoms may last for up to 2 weeks [3, 22, 28].

A summary of complications after BAE is listed in Table 9.

**Table 9:** Summary of complications [6]

Description	Grade (1–6)^a^	Reported rates (%)
Post-embolisation syndrome (pain, fever, leucocytosis)	2	1.7–31
Chest pain	2	1.4–34
Dysphagia	3	0.7–30
Subintimal dissection or perforation of the bronchial artery	3	0.3–13
Access site complication	3	3
Aortic and bronchial necrosis	4	Very rare
Phrenic nerve injury	4	Very rare
Pulmonary infarction	4	Very rare
Spinal infarction and transverse myelitis	5	0.2–6.5
Transient ischaemia, stroke, cortical blindness	5	0.6–2
Angina/myocardial infarction	5	Very rare
Respiratory failure, and death	6	

^a^Grading of complications based on the CIRSE Classification System for Complications [61]

### Mortality

If managed appropriately, the mortality at initial stay during the first episode of haemoptysis varies between 4 and 16% (7% when deaths from lung cancer excluded) [9–11]. The mortality of all causes of haemoptysis during 3 years of follow-up was 27% (20% excluding lung cancer patients), [11], whereas others reported a mortality of 22% after 5 years [47].

Patients may die because of massive haemoptysis within the first month despite BAE and surgical intervention, particularly patients with aspergilloma or due to progression of underlying lung cancer [47]. Others reported delayed death from respiratory or multi-organ failure [42]. To reduce the high mortality of patients with aspergilloma, an early intervention with repeat BAE is recommended and elective surgery should be considered thereafter [28, 47].

## Conclusion

BAE is a safe procedure for control of haemoptysis of varying aetiologies both emergent and in elective settings and possesses high rates of immediate clinical success with low complication rates. It requires a well-integrated, multidisciplinary approach.

Technical refinements of BAE have improved technical and immediate clinical success rates, and recurrences are successfully managed with multiple repeat sessions in the majority of cases. Thus, BAE also serves as a bridge to definitive therapies targeted to the underlying aetiology.

## Definitions

### Anatomic Considerations

Most commonly (70%), bronchial arteries arise from the descending thoracic aorta between the upper T5 to the lower T6 vertebral bodies, 1–2 cm above or below the carinal level [55]. Another 20% are first-order branches of the thoracic aorta or arch, but outside of the T5–T6 confines [56]. The remaining 10% originate from brachiocephalic, subclavian, internal mammary, pericardiophrenic, or abdominal (aorta, inferior phrenic, celiac) branches [21, 24, 55, 56]. In contrast to the intercostal arteries, the bronchial arteries do not run parallel to the dorsal rib segments, but rather cross them. When originating from the aorta, the branching pattern exhibits several variations [55, 57] that may not represent the four classic patterns as described by Cauldwell et al. (Table 10) [58].

**Table 10:** The four most prevalent patterns of bronchial artery anatomy [58]

Cauldwell patterns:
Type 1 (41%)—2 left, 1 right as intercostal bronchial trunk
Type 2 (21%)—1 left, 1 right as intercostal bronchial trunk
Type 3 (21%)—2 left, 2 right (one as intercostal bronchial trunk)
Type 4 (10%)—1 left, 2 right (one as intercostal bronchial trunk)
Common bronchial trunk (unknown incidence)

In the majority of cases, the right BA has a common origin with a posterior intercostal artery called the intercostal bronchial trunk (ICBT) and arises from the right anteromedial aspect of the descending thoracic aorta. In 5–10%, the variable configuration of the ICBT may have a division into the anterior spinal artery (ASA) to supply the spinal cord [13]. In this case, the spinal branch follows a typical "hairpin"-like course with a rising section that merges into the ASA after a descending intraspinal segment [56].

The upper left BA usually arises from the aorta ventrally, lateral to the carina, the lower left BA arises parallel to the superior BA, but inferior to the left main bronchus [22, 56, 57]. Systemic non-bronchial collateral arteries rather follow a transpleural course or potentially ascend via the inferior pulmonary ligament, never joining the bronchial tree [56].

### Grading of Haemoptysis

Although haemoptysis is a clearly defined symptom, there is still no consensus on the definition of SH [1, 20, 59]. The threshold for defining SH varies between 200 and 1000 ml in 24–48 h. Others assess SH in terms of its respiratory and/or haemodynamic repercussions [1] or use a scoring system [10].

Since the total volume of the conducting airways averages 150 ml in adults, any given haemorrhage in the airways can quickly become life-threatening [20].

Many still use a consensus definition that mild haemoptysis results in less than 100 ml/day or less than 50 ml/episode) [6]. Moderate haemoptysis (100–300 ml/day (≈ 1 cup)) can have a high mortality rate due to associated asphyxia and requires early treatment [20]. In severely respiratory-compromised patients (FEV_1_ < 35%), significant disturbances of the gas exchange, as well as suffocation, may occur with haemoptysis of far less than 300 ml [4, 6].

Severe, LTH - may be defined as any haemoptysis that: (1) is > 100 ml in 24 h; (2) causes respiratory failure (SpO_2_, < 60%) necessitating intubation and mechanical ventilation; or (3) causes haemodynamic instability. The cut-off volume of 100 ml per 24 h was selected because it is the smallest amount of haemoptysis that is reported in literature to threaten the life of the patient [59].

## Abbreviations

ASA: Anterior spinal artery
BAE: Bronchial artery embolisation
BC: Bronchoscopy
CT: Computed tomography
CTA: Computed tomography angiography
CF: Cystic fibrosis
CR: Chest radiograph
COPD: Chronic obstructive pulmonary disease
CPA: Chronic pulmonary aspergillosis
DC: Diagnostic catheter
DMSO: Dimethyl sulfoxide
DSA: Digital subtraction angiography
FB: Flexible bronchoscopy
FEV_1_: Forced expiratory volume in 1 s
ICBT: Intercostal bronchial trunk
LTH: Life-threatening haemoptysis
MC: Microcatheter
MDCT: Multidetector computed tomography
NBCA: *N*-Butyl-2-cyanoacrylate glue
PVA: Polyvinyl alcohol
PAVM: Pulmonary arteriovenous malformation
SH: Severe haemoptysis
TB: Tuberculosis
